# Psychotic Relapse in a Patient with Schizophrenia and Graves’ Disease-Induced Thyrotoxicosis: A Case Report

**DOI:** 10.1192/j.eurpsy.2026.12195

**Published:** 2026-07-31

**Authors:** M. E. Arévalo Gil, P. Nava García, M. Sobredo Vega, N. A. Linero Ríos, S. Orrego Molina, F. Pascual Picazo

**Affiliations:** 1 Hospital Universitario Infanta Leonor; 2 Hospital Central de la Defensa Gómez Ulla, Madrid, Spain

## Abstract

**Introduction:**

The coexistence of endocrine and psychiatric disorders poses significant diagnostic and therapeutic challenges. Thyrotoxicosis, particularly due to Graves’ disease, may exacerbate or precipitate psychotic symptoms, especially in patients with schizophrenia. This case illustrates how endocrine dysregulation contributed to psychotic relapse in a patient with chronic schizophrenia and poor treatment adherence.

**Objectives:**

To describe the clinical presentation, diagnostic complexity, and multidisciplinary management of a psychotic decompensation associated with untreated Graves’ disease in a patient with schizophrenia.

**Methods:**

A 51-year-old man with paranoid schizophrenia and type 2 diabetes was admitted involuntarily after family members reported severe behavioral disturbances at home. He had a long history of poor adherence to psychiatric follow-up and antipsychotic treatment. On admission, he presented with marked disorganization, paranoid and bizarre delusions, lack of insight and psychomotor agitation. Laboratory tests revealed thyrotoxicosis (TSH <0.01 μU/ml; free T4: 6.73→2.59 ng/dl; free T3: >20→10.4 pg/ml; TSI >40 U/L). Cardiac monitoring showed sinus tachycardia and a previously unknown left bundle branch block.

**Results:**

Antipsychotic therapy with oral paliperidone (up to 12 mg/day) and olanzapine (up to 15 mg/day, later reduced to 10 mg/day due to daytime sedation) led to progressive improvement in thought organization and behavior. Endocrinology restarted methimazole and propranolol, achieving a gradual decline in thyroid hormone levels. As endocrine parameters improved, psychotic symptoms diminished, suggesting a close temporal relationship.

Because of persistent non-adherence, severe behavioral disturbances at home, and with the family’s consent, a 6-month long-acting injectable formulation of paliperidone was initiated, despite being off-label. The patient tolerated it well, with no extrapyramidal symptoms or adverse events. Social work evaluation confirmed social vulnerability and poor support, prompting judicial notification and recommendations for protective measures. During hospitalization, the patient progressively regained collaboration, re-established contact with relatives, and showed stable behavior at discharge.

**Conclusions:**

This case highlights the bidirectional relationship between thyrotoxicosis and psychosis. Thyroid hyperactivity can exacerbate psychiatric symptoms and hinder stabilization in schizophrenia. Systematic endocrine monitoring and close collaboration between psychiatry and endocrinology are essential for optimal management in such complex cases. In cases of severe behavioral dysregulation and poor adherence, long-acting injectable antipsychotics—even off-label—may represent a safe and effective strategy when supported by family involvement and multidisciplinary consensus.

**Disclosure of Interest:**

None Declared

